# Incidence of HIV in Windhoek, Namibia: Demographic and Socio-Economic Associations

**DOI:** 10.1371/journal.pone.0025860

**Published:** 2011-10-04

**Authors:** Marielle Aulagnier, Wendy Janssens, Ingrid De Beer, Gert van Rooy, Esegiel Gaeb, Cees Hesp, Jacques van der Gaag, Tobias F. Rinke de Wit

**Affiliations:** 1 PharmAccess Foundation, Amsterdam, The Netherlands; 2 Department of Development Economics, VU University Amsterdam, Amsterdam, The Netherlands; 3 PharmAccess Foundation Namibia, Windhoek, Namibia; 4 Multi-disciplinary Research Center, University of Namibia, Windhoek, Namibia; 5 Namibia Institute of Pathology, Windhoek, Namibia; 6 Amsterdam Institute for International Development, Amsterdam, The Netherlands; 7 Brookings Institution, Washington, D.C., United States of America; 8 Department of Global Health, Academic Medical Center of the University of Amsterdam, Amsterdam Institute for Global Health and Development, Amsterdam, The Netherlands; Kenya Medical Research Institute - Wellcome Trust Research Programme, Kenya

## Abstract

**Objective:**

To estimate HIV incidence and prevalence in Windhoek, Namibia and to analyze socio-economic factors related to HIV infection.

**Method:**

In 2006/7, baseline surveys were performed with 1,753 private households living in the greater Windhoek area; follow-up visits took place in 2008 and 2009. Face-to-face socio-economic questionnaires were administrated by trained interviewers; biomedical markers were collected by nurses; GPS codes of household residences were recorded.

**Results:**

The HIV prevalence in the population (aged>12 years) was 11.8% in 2006/7 and 14.6% in 2009. HIV incidence between 2007 and 2009 was 2.4 per 100 person year (95%CI = 1.9–2.9). HIV incidence and prevalence were higher in female populations. HIV incidence appeared non-associated with any socioeconomic factor, indicating universal risk for the population. For women a positive trend was found between low per-capita consumption and HIV acquisition. A HIV knowledge score was strongly associated with HIV incidence for both men and women. High HIV prevalence and incidence was concentrated in the north-western part of the city, an area with lower HIV knowledge, higher HIV risk perception and lower per-capita consumption.

**Discussion:**

The HIV incidence and prevalence figures do not suggest a declining epidemic in Windhoek. Higher vulnerability of women is recorded, most likely related to economic dependency and increasing transactional sex in Namibia. The lack of relation between HIV incidence and socio-economic factors confirms HIV risks for the overall urban community. Appropriate knowledge is strongly associated to lower HIV incidence and prevalence, underscoring the importance of continuous information and education activities for prevention of infection. Geographical areas were identified that would require prioritized HIV campaigning.

## Introduction

Namibia, with a population of approximately 2.2 million is classified as a middle income African country; although income disparity is one of the largest in the world [1]. In 2004 more than 38% of the population was living below the poverty line [2].

With a reported national prevalence of 15.3% in the adult population of 15–49 years [3], Namibia is experiencing one of the largest HIV epidemics in Africa. Since 1996, AIDS has been the leading cause of death and contributed to a drop in life expectancy from 65 to 61 years, between 1990 and 2008^1^. Over the past two decades, the Government of Namibia has prioritized HIV and AIDS in its development undertakings. This resulted, amongst others, in impressive increase in access to antiretroviral treatment with a >75% coverage according to the new WHO guidelines [4]. This was greatly facilitated by the rapid scale up of vertical donor funding (PEPFAR and GFATM) which resulted in 28.5% of the 2008/2009 Total Health Expenditure of N$4,945/US$706 million being spent on HIV/AIDS. 51% of this amount is donor funded [5]. Recent evidence suggested that national HIV prevalence in Namibia started to decline or stabilize since 2002 [6]. However, since prevalence is still high, fighting the HIV epidemic requires continuous vigilance. Namibia is facing rapid urbanization, especially towards the capital city of Windhoek [7], which might exacerbate the HIV epidemic [1], [8]–[10] as economic inequality is heightening in city areas and has been found to be highly associated with sexually transmitted diseases and HIV [11].

Ensuring population's good health presents many challenges within the complex urban context of developing countries [9], [12]–[14]. This paper presents HIV incidence and prevalence estimates from household surveys conducted in Windhoek-Namibia between 2006/7 and 2009. Associations with HIV incidence are explored with demographic and socio-economic factors, which may contribute to guiding future prevention and control efforts in the capital city of Namibia.

## Methods

### Ethics Statement

The study was approved by the Research Ethics Committee at the Namibian Ministry of Health and Social Services (MOHSS). Anonymity of HIV test results was safeguarded through specific bio-medical protocols that linked the results to the household survey dataset using anonymous identification numbers. Participants' names and addresses were kept separate from the data. Individuals who wished to know their test results were referred to existing VCT centers in Windhoek, since individual HIV results cannot be based on a single test as performed during the current surveys.

### Study design and target population

In 2004, a project was started in Namibia named “Okambilimbili” (“Butterfly”) which stimulated the development and implementation of low-cost voluntary basic health insurances for low income workers, by temporarily subsidizing medical aid (health insurance) premiums for low income workers and creating a risk-equalization fund for HIV and AIDS [15], thus improving access to affordable health care and reduction of risk of catastrophic healthcare expenditures. In order to evaluate the project's impact, large-scale household surveys were implemented in a random sample of the greater Windhoek area in 2006/7 and re-conducted in 2008 and 2009. These surveys measured health indicators, biomedical markers and socio-economic characteristics in Namibian households.

The sample design for the original survey was a representative stratified two stage probability sample. The first stage consisted of geographical areas (primary sampling units (PSU), as defined for national census purposes) from Windhoek and the second stage consisted of randomly selected households in these areas. Target population consisted of private households in the greater Windhoek area, excluding people in hospitals, hostels and prisons. Surveys were conducted with the same households in 2006/7, 2008 and 2009. Households that had relocated their residence between surveys were traced wherever possible; field workers revisited the households a minimum of 3 times to contact absentees.

### Data collection

#### Socio-economic questionnaire

Face-to-face questionnaires were administrated by trained interviewers from the Multi-disciplinary Research and Consultancy Centre (MRCC) of the University of Namibia, after household consent. The demographic and socio-economic questionnaire collected information on gender, age, education level, employment status, income, household composition, housing characteristics, household consumption, etc. In addition the survey collected specific information on health (preventive and reproductive health, chronic diseases, illness and injury, hospitalizations), healthcare spending and health behaviour (alcohol consumption, smoking, contraception use, age at first sexual intercourse, HIV testing, etc.). Information regarding children under 12 years old was obtained from a household adult; information from adolescents 12–18 years old was collected by direct interviews after consent from their parents.

#### Biomedical markers

Biomedical markers were collected by qualified nurses. Anonymous HIV tests were performed on participants aged 12 years or older who(se parents) provided informed consent. HIV screening was performed on non-invasive oral fluid samples collected with OraSure® HIV-1 Oral Specimen Collection Device (OraSure Technologies, Inc., Bethlehem, PA). Samples were labelled with a barcode that corresponded to the barcode on the survey questionnaires and shipped to the Namibia Institute of Pathology (NIP). At NIP a HIV test was performed using the Oral Fluid Vironostika HIV Uni-Form II Micro-ELISA (bioMérieux Inc., Durham, NC). This procedure had previously been validated and approved by the MOHSS for use in anonymous epidemiological surveys [16]. HIV test results from NIP could thus be linked to the household survey forms but not to particular individuals, since the list of their names and corresponding barcodes was kept separate at MRCC and combination of information with NIP was not possible. HIV test results were not shared with survey participants; those who wished to know their HIV status were referred to the appropriate facilities for HIV testing according to the national protocol.

#### Household socio-economic status and welfare

Household consumption was measured to investigate household socio-economic status and welfare. Annual household consumption was calculated by summing weekly food consumption multiplied by 52, monthly consumption of regular non-food items multiplied by 12 and annual consumption of remaining non-food items in each household. Subsequently, per capita consumption was calculated by dividing annual household consumption by the number of members in each household [17]. Individuals were assigned to a consumption category based on their per capita consumption level in comparison to the poverty line (264 N$/38US$ per month in the 2003 Namibia Household Income and Expenditure Surveys [2]), corrected for inflation. The poverty line was calculated at 302 N$/43 US$ per month in 2006 and 373 N$/53US$ per month in 2009. The consumption categories were defined as follows: 1) below poverty line, 2) between poverty line and twice the poverty line, 3) between twice and three times poverty line and 4) more than three times poverty line.

#### HIV knowledge and risk perception score

An HIV knowledge score was constructed by summing all questions on HIV knowledge in the questionnaire (see Appendix S1 for more information). Correct responses were scored 1 and incorrect responses 0. The score ranged from 0 to 11. The personal HIV risk perception score was constructed using the following question: “what do you think your chances are of getting HIV/AIDS?”. The response score were No risk at all (0), Small (1), Moderate (2), High (3), Don't know (4). The individuals who refused to answer were eliminated from the analyses.

### Data analysis

#### HIV incidence

**Figure 1:**
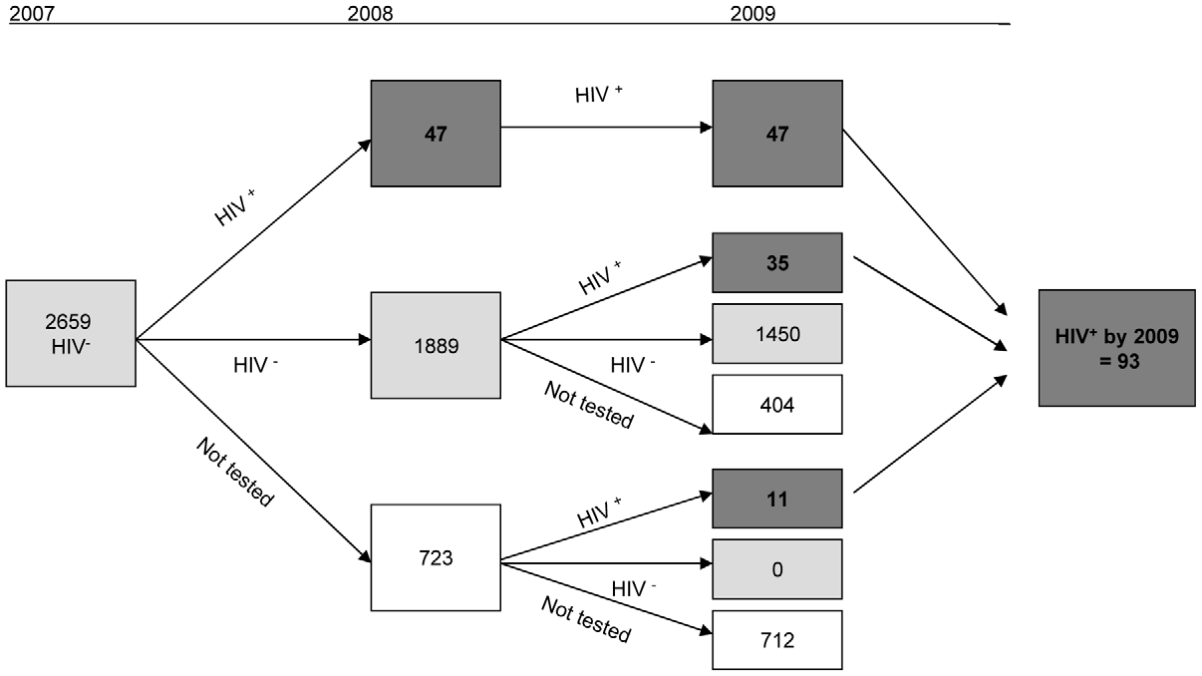
Follow up of individuals aged 12 years or older, who participated to the baseline survey and were seronegative at baseline (2007).

Incidence rate was defined as number of new HIV infections per 100 person-years (PY). Person-years were based on all participants aged 12 years or older in 2006/7 uninfected at baseline and followed up until round 3 of the survey (Figure 1). Individuals who acquired HIV infection were assumed to have been infected halfway through the observation period. The calculation does not account for the window period of infection. This will not affect the estimates assuming a constant HIV incidence rate over the years 2007–2009.

#### Factors associated with HIV status and geographical analysis

Simple logistic regressions were used to study demographic and socio-economic factors associated with HIV incidence separately in males and females, older than 18 years. Variables with p<0.15 were introduced into backward multiple logistic regression (exit threshold p>0.15). Statistical analyses were performed with SPSS, version 13.0 (SPSS Inc., Chicago, 2002). We considered that variables were significant at p< = 0.05 and that 0.05<p<0.10 indicated a trend at the limit of significance.

For each PSU three sets of latitude/longitude coordinates were recorded using hand-held GPS devices. The geographical centre of each PSU was calculated by averaging its latitudes/longitudes to obtain the arithmetic mean. Subsequently, data was fed into QlikView 9.0 (Qliktech, Radnor, Pennsylvania) and superimposed on Google Maps (Google Inc, Mountain View). Each bubble represents a PSU with the size of the bubble proportional of the scores. Because of the small incident HIV sample in each PSU, the incidence map is not shown in the current manuscript.

## Results

### Socio-economic characteristics

The original baseline survey sample aimed at 2,000 households and realized a voluntary participation of 1,753 (88%) in the socio-economic part of the questionnaire in 2006. The households were again visited in 2008 (1,154 households i.e. 66% of baseline household participants) and 2009 (861 households i.e. 75% of 2008 household participants). Three years after baseline, 56.5% of the males and 57.7% of the females still participated. Attrition was mostly due to mobility of the target population and refusal to again participate. Eight hundred and sixty one (861) households representing 3,168 individuals aged 12 years or older in 2006 participated in all three rounds of survey. The mean household size was 4.8 persons in 2006 (Table 1). Households lost to follow-up were slightly younger, more affluent and with higher education level (data not shown).

**Table 1:** Socio-economic characteristics of individuals (≥12 years) who participated in the 3 rounds of surveys (n = 3,168) and comparison with the Namibian population [44].

Category	Subcategory	Population (> = 12 years)		Namibian population (DHS 2006/07)	
Household size	*(mean)*	4.8		4.5	
Gender	Female	51.8%		53.0%	
Age	*(mean)*	30.3 (+/−13.3)		NA	
		Females (>15 y)	Males (>15 y)	Females (15–49 y)	Males (15–49 y)
Marital status	Never married	65.1%	64.2%	57.9%	65.0%
	Married/Union	30.2%	34.7%	35.2%	30.8%
	Divorced/separated	1.8%	0.9%	4.4%	3.9%
	Widowed	2.9%	0.1%	2.6%	0.3%
		Females*	Males*	Females (20–49 y)	Males (20–49 y)
Age at first sexual intercourse	*(median)*	19.0	18.0	18.9	18.0
		Females (>5 y)	Males (>5 y)	Females (>5 y)	Males (>5 y)
School grade	None	7.9%	11.1%	14.6%	15.6%
	Primary	9.5%	14.7%	41.6%	44.7%
	Secondary	79.8%	70.7%	37.5%	33.3%
	Higher	2.8%	3.5%	4.7%	4.9%
Employed during the past year (>15 years)		47.4%		NA	
Per capita consumption level	<302 N$/month	19.6%		NA	
	302–603 N$/month	26.3%			
	604–905 N$/month	15.9%			
	≥906 N$/month	38.2%			
Covered by health insurance policy		22.8%		NA	
Personal HIV perception risk, 2008	No risk at all	47.1%		NA	
	Small	14.3%			
	Moderate	7.8%			
	High	4.5%			
	Don't know/refused	26.4%			
HIV knowledge score, 2009	*(mean)*	5.2 (+/−4.7)		NA	

*Among individuals aged >12 years who had sexual intercourse; NA: Not available.

Among the sample, 51.8% were females and the mean age in 2006 was 30.3 years (standard deviation (sd) +/−13.3). Nearly 10% of individuals of school-age (>5 years) never attended school, 12.0% had a pre-school or primary level, 75.4% a secondary level and 3.1% a higher level. About half (47.4%) of the people aged 15 years or older did work during the past 12 months. Nearly one fifth (19.6%) of the individuals declared a consumption level below the national poverty line (302 N$/month) and 22.8% subscribed for a health insurance policy in 2006.

### HIV knowledge, attitudes, practices and risk perception

Among individuals who declared to ever have had sexual intercourse, the mean age for first sexual intercourse was 18.8 years (sd+/−3.5). The mean age for sexual debut was 19.2 years (sd +/−3.4) in females and 18.6 years (sd +/−3.7) in males (p = 0.02). In the population aged 15 years and older in 2006, 32.4% were living in couple (married or consensual union) and 64.6% were never married.

Nearly half of the individuals (47.1%) perceived themselves to be not at risk for HIV infection in 2008, 14.3% at low risk, 7.8% at moderate risk and 4.5% at high risk. Younger people (<25 years) were more likely than older ones to view themselves as being not at risk (50.0% vs 45.6%, p<0.001). The mean score for HIV knowledge was 5.19 (sd+/−4.7). This score was significantly higher in population aged 25 years or older than in the younger population (5.6 vs 4.5, p<0.001).

### HIV incidence

The incidence analysis includes all individuals aged 12 years or older who tested HIV negative at baseline in 2006/2007 and for whom HIV status was known in 2009. Where relevant, HIV positive status was prospectively extrapolated for missing test results in future survey rounds. HIV negative status was retrospectively extrapolated for missing test results in prior survey rounds. Twenty-two individuals tested positive in 2006/7 with borderline OD-values < 2 times the cut-off point and tested negative in the second independent HIV test. The results of the second test were used in this analysis. This yields a baseline sample of 2,659 HIV negative individuals. For 1,543 of the initially HIV negative people, HIV status is known in 2009 (Figure 1).

Incidence estimates were based on a total of 3,858 person-years of follow up on people aged 12 years or older in 2006. During the follow-up period, 93 individuals had acquired HIV infection by 2009, resulting in an overall incidence rate of HIV infection of 2.4 per 100 PY (95%CI = [1.9–2.9]). The incidence rate was lower in male populations (1.6; 95%CI = [1.0–2.1] per 100 PY) than in female population (2.6; 95%CI [2.0–3.3] per 100 PY) (Table 2). HIV incidence was lower in the youngest age group (12–24 years) among both sexes: 0.8 per 100 PY in males and 1.9 per 100 PY in females. The mean age of women who became HIV positive by 2009 was 31.8 versus 36.6 in men population (p = 0.06).

**Table 2:** HIV incidence and HIV prevalence estimates by age and sex.

	INCIDENCE^1^ 2007–2009			PREVALENCE^2^ 2009		
	N	HIV incidence (per 100 PY)	95%CI	N^1^	HIV prevalence (%)	95%CI
**Population > = 12 years**	**1543**	**2.4**	**[1.9–2.9]**	**2119**	**14.6**	**[13.1–16.1]**
Males	760	1.6	[1.0–2.1]	947	13.4	[11.2–15.6]
12–24 years	319	0.8	[0.2–1.4]	377	6.6	[4.1–9.2]
≥25 Years	441	2.2	[1.3–3.1]	570	17.9	[14.8–21.1]
Females	958	2.6	[2.0–3.3]	1172	15.6	[13.5–17.7]
12–24 years	368	1.9	[1.0–2.7]	401	6.2	[3.9–8.6]
≥25 Years	590	3.1	[2.2–4.0]	771	20.5	[17.7–23.4]
**Population 15–49 years**	**1290**	**2.4**	**[1.9–3.0]**	**1623**	**15.8**	**[14.1–17.6]**

PY: Person-years; CI: Confidence interval;

1 Sample population: individuals aged 12 years or older in 2007, who participated to the baseline survey and were seronegative at baseline.

2 Sample population: persons who participated to the survey in 2009 and accepted to perform a test.

### HIV prevalence

In 2006/7, the overall rate of HIV-infection was 11.8% among the participant aged 12 years or older and 13.5% among participants aged 15–49 years. In 2009, of the 3,787 individuals aged 12 years or older who participated in the survey 2,119 accepted to perform an HIV test. The overall HIV prevalence was 14.6% (95%CI = [13.1–16.1]) in this population and 15.8% (95%CI = [14.1–17.6]) in the population aged 15–49 years (Table 2).

The HIV prevalence was higher among females (15.6%; 95%CI = [13.5–17.7]) than among males (13.4%; 95%CI = [11.2–15.6]) and concerned more frequently people aged 25 years or older (Table 2). For females, HIV prevalence peaks in the 25–39 age-group but for men it continues to be high after 40 years old.

### Factors associated with HIV incidence

For adult women (>18 years old), very few socio-economics factors were significantly associated with HIV incidence in univariate analyses (Table 3): younger age at 1^st^ sexual intercourse (p = 0.07; borderline significant) and lower consumption level (p = 0.01). There was no evidence for difference in HIV incidence by marital status, school grade, employment status, health insurance subscription and number of sexual partners declared in the past year. HIV acquisition was significantly less frequent among women with higher HIV knowledge score (p<0.001). Multiple logistic regression indicated that only HIV knowledge score remained associated with incident HIV (p<0.001). Trends with lower consumption level were observed (p = 0.12) (Table 3).

**Table 3:** Socio-demographic characteristics associated with HIV incidence between 2007 and 2009 among women and men aged 18 years or older^1^ – Univariate and multiple analysis.

		*WOMEN*							MEN						
			Univariate analysis			Multiple analysis				Univariate analysis			Multiple analysis		
*Characteristics in 2006*		n (749)	OR	95%CI	P	OR	95%CI	P	n (580)	OR	95%CI	p	OR	95%CI	p
*Age*		749	0.98	0.97–1.01	0.15	–	–	**–**	580	1.02	0.99–1.05	0.12	–	–	–
*Marital status*	Never married	328	1		0.60				243	1		0.73			
	Married	216	0.69	0.36–1.33					198	1.37	0.57–3.29				
	Consensual union	82	0.65	0.24–1.72					75	1.66	0.55–5.03				
	Divorc./sep./widow	52	1.01	0.39–2.86					11	2.33	0.27–20.0				
*School-grade*	None	55	1		0.63				61	1		0.79			
	Primary	49	0.74	0.12–4.61					54	0.82	0.17–3.91				
	Secondary	465	1.24	0.36–4.19					333	0.42	0.19–1.97				
	Tertiary	25	2.36	0.44–12.6					27	0.88	0.19–6.63				
*Employment status, past year*	Employed	418	0.86	0.49–1.52	0.60				369	1.94	0.72–5.21	0.19			
	Unemployed	258	1						158	1					
*Per capita consumption level*	<302* N$/month	116	1			1			85	1					
	302–603 N$/month	151	1.1	0.50–2.10	**0.01**	0.80	0.34–1.89	**0.12**	130	1.05	0.33–3.20	0.82			
	604–905 N$/month	99	0.36	0.13–1.02		0.52	0.17–1.62		72	0.94	0.24–3.64				
	≥906 N$/month	275	0.30	0.14–0.85		0.36	0.15–0.89		224	0.61	0.16–2.40				
															
*Health Insurance policy*	No	484	1						370	1					
	Yes	196	1.18	0.75–2.83	0.27				162	0.87	0.38–1.98	0.73			
*Age at 1^st^ sexual intercourse*		613	0.91	0.82–1.01	**0.07**	–	–	–	427	0.92	0.89–1.12	0.92			
*Personal HIV risk perception*	No risk at all	329	1		**0.08**	–	–	–	205	1		**0.08**	–	–	–
	Small	83	1.25	0.4–3.5					80	3.2	0.9–10.9				
	Moderate to high	85	2.60	1.1–6.					56	3.9	1.3–11.4				
	Don't know	144	2.11	1.0–4.4					115	3.8	1.1–14.1				
*HIV knowledge score, 2009*		749	0.73	0.67–0.80	**<0.001**	0.74	0.64–0.78	**<0.001**	580	0.73	0.65–0.83	**<0.001**	0.74	0.65–0.85	**<0.001**

1 Sample population consist of all individuals with HIV negative status at baseline and a known HIV status in 2009 (ie individuals who performed a test in 2009 and individuals tested positive in 2008 but did not perform a test in 2009) - see Figure 1. – TB: tuberculosis – * poverty line in 2006.

Among adult men, there was little evidence that HIV acquisition was associated with any socioeconomic factor. Only personal HIV risk perception (p = 0.08; borderline significant) and HIV knowledge score (p<0.001) were associated with HIV incidence. In multiple logistic regression, HIV knowledge score remained the only significantly association with HIV acquisition (Table 3).

### Geographic distribution

**Figure 2:**
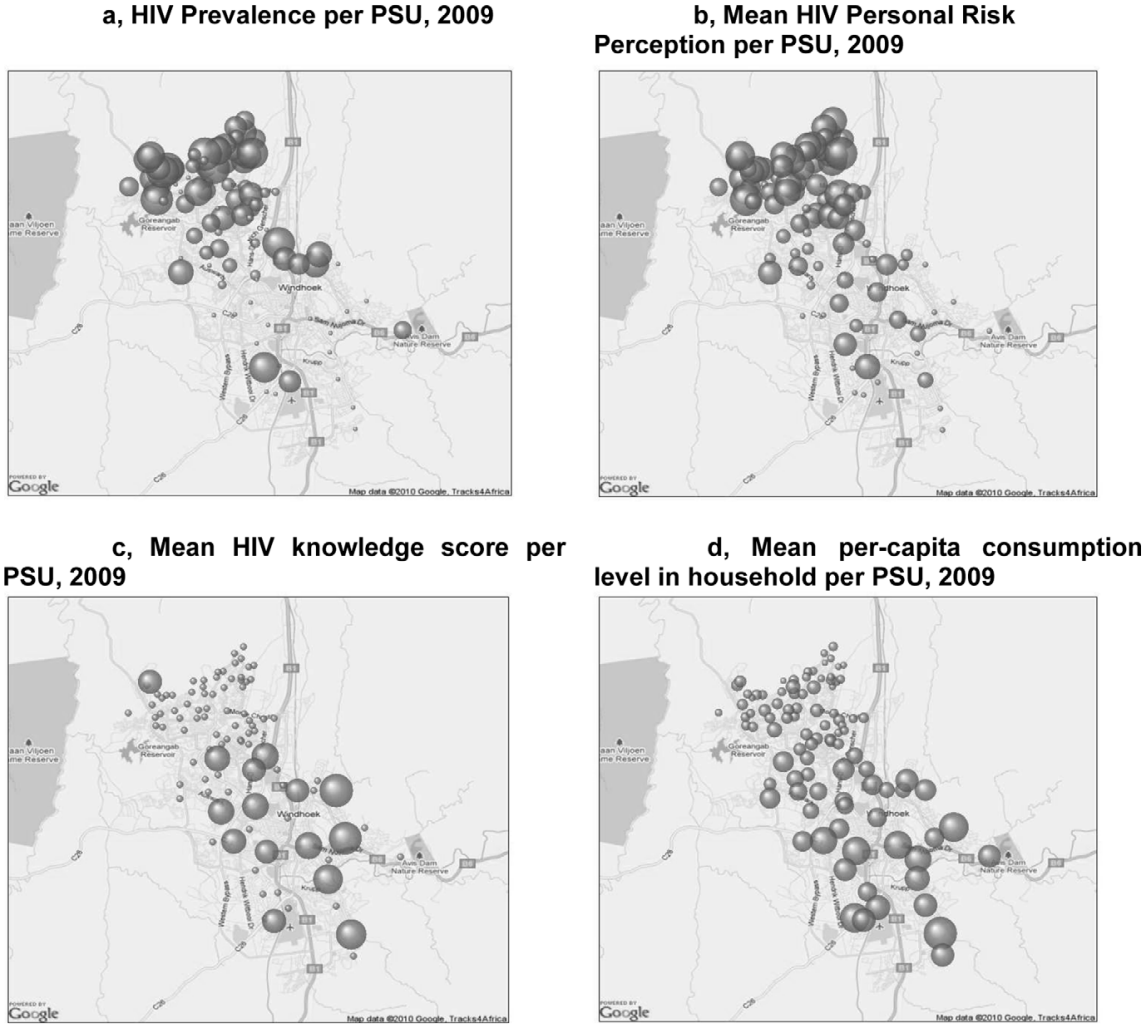
Geographic representation of the city of Windhoek with “bubble-graphs” representing values of various indicators. Figure 2a, HIV Prevalence per PSU, 2009/The HIV prevalence ranged from 0 to 47%. Figure 2b, Mean HIV Personal Risk Perception per PSU, 2009: The HIV risk perception was measured on a 1 to 4 scale. The mean score per PSU ranged from 0 to 1.7. Figure 2c, Mean HIV knowledge score per PSU, 2009: HIV knowledge was measured according to a 1–11 scale. The mean score per PSU ranged from 2.3 to 11.0. Figure 2d, Mean per-capita consumption level in household per PSU, 2009: Mean Per capita consumption level per PSU ranged from 485$ to 125,800$ per month. One outlier of 530,800$ per month has not been included in this graph.

The geographic distribution per PSU of HIV prevalence, HIV personal risk perception, HIV knowledge score and mean per-capita consumption in the Greater Windhoek Area in the year 2009 are represented in Figures 2a–d. It is noticeable that high HIV prevalence is concentrated in the north-western part of the city (Figure 2a). These areas are also characterized by smaller HIV knowledge scores (Figure 2b), higher personal HIV risk perception (Figure 2c), and lower per-capita consumption (Figure 2d).

## Discussion

This is the first paper reporting on HIV incidence in Windhoek, Namibia as determined by three consecutive household surveys (2006–2009). A high HIV incidence was observed in Windhoek in both sexes: 2.6 per 100 PY in females and 1.6 per 100 PY in males. The female HIV incidence in Windhoek appeared comparable to data reported from urban adult population in Zambia and Zimbabwe but lower than data reported in Malawi or South Africa [18]–[20]; for men the HIV incidence was lower than the data observed in the only one incidence survey performed in adult urban men in Kenya [21].

The overall HIV prevalence in this study for the age group 15–49 years was 15.8% (95%CI = [14.1–17.6]). This is similar to the most recent statistics published by WHO for Namibia (15.3% in 2007) in the same age group [3] but higher than the latest estimation [22] published recently for 2008/2009 (13.3%). Moreover, the HIV prevalence data in Windhoek confirm the general observation that women acquire HIV infection on average 5 years earlier than men and that prevalence in younger women (≤25 years) is higher than in the corresponding male age group [23]. It is underscored that age and sex differentials in distribution of HIV infection remain key drivers of generalized epidemics, highlighting the vulnerability of young women. One other reason of HIV proliferation in women population might be the increasingly acceptable form of transactional sex in Namibia [24]. Research demonstrated that “transactional sex among women was associated with a 54% increase in odds of being HIV seropositive” [25] and concern more often the poorest communities who want to achieve higher socioeconomic status [25]–[28]. This result confirms the trend found between women wealth status and HIV acquisition [1], [29].

Recent evidence suggests that HIV prevalence is declining since 2002 in Namibia [6]. However, the current survey in Windhoek does not substantiate this finding. In fact, the Windhoek HIV prevalence appears to remain stable: in the order of 15%. It is possible that with a >75% coverage of HIV patients by antiretroviral therapy in Windhoek, more HIV positive patients remain alive and thus contribute to increased overall HIV prevalence estimations.

The data presented in this manuscript are collected through household surveys, a methodology that was accepted by the WHO to be more representative of a population [30]. Careful analysis of our survey data led to the observation that individual surveyors can contribute to data aberrations, but these cases were excluded from the current analyses [31]. Therefore, it can be stipulated that the current Windhoek HIV incidence and prevalence data are the most recent and most representative to date.

With respect to the representativeness of the Windhoek data, several factors should be taken into consideration that could introduce biases. Most importantly, data may be influenced by the attrition rate of 43% over two years. However, very similar attrition rates are observed in other three year follow-up surveys in African settings [32]–[35] and therefore our data should be comparable to the literature. In line with the literature [33], non-response rates for HIV testing in the sample tends to be higher among the wealthier, better educated and younger households. The wealthier and higher educated population in Windhoek has lower HIV prevalence [36]. Therefore HIV incidence and prevalence might be overestimated. Previous research has indicated that correction for non-response based on such observed characteristics tends to have small and insignificant effects on HIV prevalence estimates [37]–[38]. In contrast, recent evidence in Namibia suggests that the bias due to *unobserved* characteristics may be significant and that non-respondents may be two to three times more likely to be infected than respondents [39].

Thus, on the one hand the attrition of the relatively more affluent survey participants may lead to upward estimations of overall population HIV incidence and prevalence. On the other hand, attrition may have been predominated by participants with a higher risk of HIV infection. Based on the latter, HIV prevalence would be underestimated. If these factors would balance each other out and with the currently estimated HIV incidence of 2.4%, we can estimate HIV prevalence including those individuals who were HIV negative in previous surveys but who did not participate in the last (2009) survey. This calculation yields an estimated overall cumulative HIV prevalence of 16% over the full three years of surveying in Windhoek.

Lastly, while prospective cohort studies are considered the “gold standard” approach to establish determinants of HIV incidence, they remain subject to biases [20] since these determinants are based on self-reported behaviours. Individuals' reports may not reflect actual behaviour because of memory bias or social desirability bias, especially when considering attitudes and behaviours regarding sexual activity or HIV [40]. Our surveys are based on actual HIV test results and are more reliable in this respect.

It has to be emphasized that this survey was performed in an urban area, characterized by persistent migrations and strong economic inequalities. It is known that HIV prevalence is higher among urban residents compared to rural populations [1], [8] and strongly associated with migrations [41]. Therefore, the HIV prevalence and incidence is likely to be lower in other parts of Namibia, probably with the exception of the Caprivi strip. Continued high urban HIV incidence in Namibia might also be partly explained by the high income disparity in Windhoek. Economic inequality was found to be highly associated with sexually transmitted diseases and HIV [11] and has been demonstrated to worsen health outcomes across all economic strata in society [42].

The geographical analyses in this paper highlight the concentration of HIV in the North-West (NW) part of the city. This salient finding is not easily picked up by statistical analyses alone. These are known to be the lower income areas occupied largely by previously disadvantaged Namibians, often migrants from within and outside Namibia. People in these areas reside more closely in informal housing, without the same access to amenities as the high/middle income areas in the South-East (SE). Thus, in Windhoek, HIV prevalence appears at least geographically to coincide with areas with poorer living standards. However, bivariate and multivariate analyses of HIV incidence did not reveal significant relations. This may be because power to detect any association was somehow lower for incidence analysis or because the factors driving the geographical correlation between poverty and HIV infection have changed over time, e.g. from economic to knowledge-related mechanisms.

The lack of a relationship between HIV incidence and socio-demographic factors such as marital status, education level, employment, etc. confirmed once again that HIV risks concern all economic strata in African urban societies [20]. Continued efforts are required to combat the epidemic at the general population level. In this respect, it is clearly demonstrated by the current survey that the only socio-demographic factors that remain significantly associated with HIV incidence as well as prevalence are related to proper knowledge of the disease (knowledge scores) and an adequate perception of the risk of HIV infection. The geographical analyses confirmed these observations by indicating correlations between HIV knowledge, HIV risk perception and HIV prevalence. It is obvious that individuals with better HIV knowledge can engage themselves in safer behaviours [43]. For this reason, the current paper is a call for increased HIV information and education prevention campaigns in Windhoek, with a particular focus on the NW part of the city. The campaign should cut across all socio-economic strata of society, focus on women and impact should be measured by continued representative HIV incidence surveys.

## Supporting Information

Appendix S1 **Construction of the HIV Knowledge score.**
(DOC)
