# Supplementary material for: Incidence of HIV in Windhoek, Namibia: Demographic and Socio-Economic Associations
Source: PLoS One. 2011 Oct 4;6(10):e25860. doi: 10.1371/journal.pone.0025860 (PMC3186802; doi:10.1371/journal.pone.0025860)
Supplement: Appendix S1 — Construction of the HIV Knowledge score. (DOC) [file pone.0025860.s001.doc]

**Appendix I: construction of the HIV Knowledge score**

1. Can people get HIV/AIDS from mosquito bites? (No=1, Yes=0, Don’t know=0)
2. Can people get HIV/AIDS by sharing food with a person who has HIV/AIDS? (No=1, Yes=0, Don’t know=0)
3. Is it possible for a healthy looking person to have HIV/AIDS? (No=0, Yes=1, Don’t know=0)
4. Could you get infected with HIV if you are exposed to: (Yes you could=0, No you could not=1, Don’t know=0)
   1. the spit of a person living with HIV/AIDS?
   2. the sweat of a person living with HIV/AIDS?
   3. The faeces of a person living with HIV/AIDS?
5. Your child could or could not get infected with HIV if he/she plays with a child who has HIV/AIDS? (Yes he/she could=0, No he/she could not=1, Don’t know=0)
6. You could or could not get infected if you care for a person living with HIV/AIDS? (Yes you could=0, No you could not=1, Don’t know=0)
7. What can someone do to reduce the risk of contracting HIV? (several responses possible) (Abstain from sex=1, Use condom=1, Limit sex to one partner=1, Other=0)
